# Exploration of the Effects of Telerehabilitation in a School-Based Setting for At-Risk Youth

**DOI:** 10.5195/ijt.2017.6215

**Published:** 2017-06-29

**Authors:** SARA BENHAM, VARLEISHA GIBBS

**Affiliations:** 1 DEPARTMENT OF OCCUPATIONAL THERAPY, UNIVERSITY OF THE SCIENCES IN PHILADELPHIA, PHILADELPHIA, PA, USA; 2 DEPARTMENT OF OCCUPATIONAL THERAPY, WESLEY COLLEGE, DOVER, DE, USA

**Keywords:** Children, Exercise program, Pediatrics, School-based, Telerehabilitation

## Abstract

This study explored the efficacy and feasibility of a motion-capture program that may be utilized for telerehabilitation purposes. Two children attending a school for at-risk children received 20 sessions of Timocco, with sessions lasting for 30 minutes, under the guidance of research assistants. The researchers employed a mixed methods design to analyze quantitative data and qualitative interviews. Both children improved their fine and gross motor coordination, as measured on the Bruininks-Oseretsky Test of Motor Proficiency Short Form. To explore feasibly, qualitative verbal reports of the child participants and research assistants were obtained. The children and research assistants reported positive experiences with the utilization of this platform. A collaborative, consultative telerehabilitation model may be a promising delivery mode of services for improving motor skills in children, with frequent input from the child, aide or teacher, and clinician. Further exploration is needed for telerehabilitative service delivery for at-risk children.

Therapy practitioners utilize the term “telerehabilitation” to specify virtual service deliveries (Gibbs & Toth-Cohen, 2011). While improving access to quality health care, this two-way interactive telecommunication technology decreases the geographic and temporal constraints individuals may have by allowing them to remain in their natural environments to receive therapeutic services. Telerehabilitation is used increasingly to improve access to services for children in rural areas, which reduces barriers of travel to subspecialty care. Families may also receive the same benefits when choosing to educate their children through online charter schools, allowing children to learn at a self-directed pace and outside of normal classroom hours (Ahn, 2011). Not only is this form of virtual communication a solution to improve educational and therapeutic service access to children in rural areas, video teleconferencing may provide low-cost alternatives for screening services in urban areas (Ciccia, Whitford, Krumm, & McNeal, 2011). Studies from both rural and urban environments have reported high satisfaction rates from consumers across the lifespan (Ashburner, Vickerstaff, Beetge, & Copley, 2016; Criss, 2013; Schein, Schmeler, Saptono, & Brienza, 2010; Tsaousides, D’Antonio, Varbanova, & Spielman, 2014)

Published evidence suggests that telerehabilitation programs have the potential to improve functioning in individuals with motor coordination impairments, a frequently cited reason for therapy referral. A pilot study was conducted on children ages 6 to 11 years with motor skill impairments affecting their handwriting performance for school-based activities (Criss, 2013). Results indicated fine motor and handwriting improvements, as well as high satisfaction rates, in the majority of participants. In another pilot study, videogame telerehabilitation was used with adolescents with hemiplegic cerebral palsy. All subjects demonstrated improvements in hand motor skill (Golomb et al., 2010).

However, the use of telerehabilitation is not without limitations. Within rural environments, some participants report frustration with technical difficulties (Ashburner, Vickerstaff, Beetge, & Copley, 2016; Schein, Schmeler, Saptono, & Brienza, 2010). Furthermore, overutilization of remote technology poses a risk by replacing in-person contact. With this service delivery model still in the developmental stages, there is much uncertainty for the consumer and service provider. Coupling this notion, along with the exploration of telerehabilitation programs with specific consumer groups such as an at-risk population, prompts further study into effective telerehabilitation models.

The operational definition of “at-risk children” are those youth whose home environment, or circumstances of a single parent home, can lead to disadvantages in reaching developmental milestones related to skills needed in the academic environment (Davis, Kerr, & Robinson Kurpius, 2003). Conversely, such children overcome obstacles when provided with the support of service providers qualified to enhance access to appropriate care (Ungar, Liebenberg, Armstrong, Dudding, & van de Vijver, 2013). To this end, school-based therapists are positioned to enhance skills, habits, and overall life success for at-risk youth. Yet, this is an area requiring additional investigation (Weiner, Toglia & Berg, 2012). We aim to further inform the provision of school-based therapy, specifically occupational therapy (OT) services, to at-risk children.

A research gap exists related to school-based services within the private school arena. Recent development of private and charter schools for this population indicates a need for research in specialized school-based therapy. Traditional service models include OT as a mandated service for qualifying disabled students in the public school sector (United States Department of Education, Office for Civil Rights (OCR), 2015). Children who do not qualify as disabled, yet require additional support and accommodation, should receive services under a 504 plan (i.e., Section 504 of the Rehabilitation Act of 1973). However, within the private school arena, these provisions are limited as outlined in the federal guidelines. The guidelines do not mandate a comparable level of rehabilitative service and support for children in private (versus public) schools (Doran, 2015).

Internet-based programs are a practical option for the delivery of therapeutic services via telerehabilitation. Incorporating a structured consultative and collaborative model between the therapist and school staff provides a format for successful school-based programs (Donica, 2015). The OT may be able to prescribe a recommended frequency and duration of participation for the programming, with minimal oversight from aides or teachers. Despite lack of formal staff training with therapeutic activities or sensory strategies, the therapist and school staff can communicate about program adherence, and the child’s progress is enhanced.

We explored a potential methodology through the use of an internet-based, motion capture, interactive virtual gaming program, called Timocco. Timocco promotes the development of an array of cognitive and motor skills for children ages three to 10+ years. We were interested in the use of Timocco as a therapeutic service to promote the development of the aforementioned skills, the customization of the program to meet the needs of a specific, “at-risk” pediatric population in a school-based setting, and the feasibility to provide individualized, increased frequency of therapy without requiring a one-on-one time commitment of a therapist. We utilized Timocco to address the following research question: Does telerehabilitation programming in school-based settings improve gross and fine motor skills, as measured on the BOT-2, in children ages 3 to 12 years? The primary purpose of this study was to explore the efficacy of Timocco in a school-based setting. The secondary purpose was to explore such feasibly, through the qualitative verbal reports of the research assistants and the student participants.

The Institutional Review Board of the University of the Sciences in Philadelphia approved this study.

## METHODS

This investigation implemented a mixed methods case report research design (Kratochwill et al., 2013). The design allowed for exploration of this unique sample. The pre and post testing further defined the methodology as an A-B design.

## STUDY PARTICIPANTS

Two children with documented needs for occupational therapy services participated in this exploratory mixed-methods study. Recruitment occurred at a scholarship-based private school, with school admission requirements including that all households demonstrate an economic need, as well as a single-parent home (Christina Seix Academy, 2016). Therefore, we identified all students attending the school as “at-risk children” as per the operational definition. Direct OT services were not offered at the school. The school was in process of developing a consultative model for OT service delivery. Child participants were included in the study based on the following criteria: (1) children between the ages of 3–12 years; (2) received a past OT referral from the school’s director and an OT evaluation. Based on the criteria, the school’s director of health and wellness recommended two children. Table 1 depicts the two participants’ demographic and clinical characteristics.

At the recommendation of school staff, both children had received an OT evaluation prior to initiation of this study As a result, they qualified for consultative OT services within the school setting. Their evaluations indicated concerns with both fine and gross motor coordination, visual motor/perception, strength, and stability. Such areas appeared to impede their performance within the school based setting. Child #1 had significant challenges with school-based activities such as fine motor tabletop tasks. In addition, he presented with low muscle tone and poor gross motor coordination during play and physical education activities. Child #2 also presented with challenges in fine motor and tabletop activities required in the classroom setting. He presented with poor gross motor coordination as seen in frequent falls and challenges maneuvering his environment.

## INTERVENTION

### TIMOCCO GAMING SYSTEM

**Figure 1:**
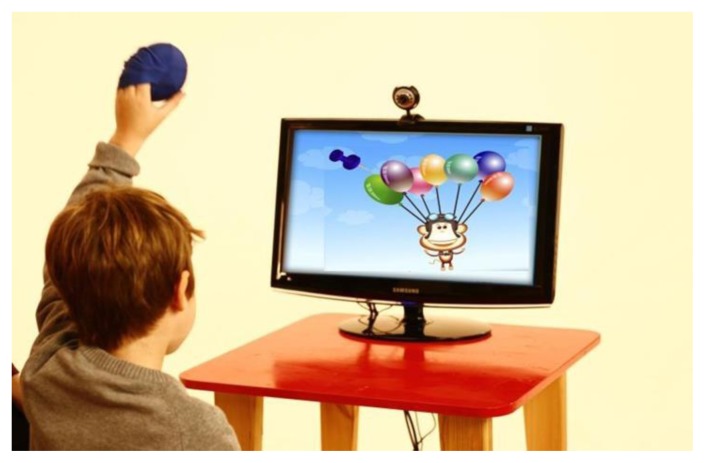
Timocco game set-up. The webcam is tracking the motions of the child by capturing the image of the gaming ball. In this image, the child’s goal is to virtually “pop” the balloons with the “push pin,” on the screen, which moves in real-time as the child moves his left upper extremity.

The Timocco program (http://www.timocco.com/) is internet-based software that can be used on a standard Mac or PC, anywhere with internet access. Hardware includes a webcam that connects to the computer by USB which detects the child’s movements by motion-capturing the provided red, blue, or green gaming balls (Figure 1). The gaming balls include a mitten feature with the availability to be slipped over a child’s hands who may have limited grasp skills. The 50+ games were designed by an OT, and can be quickly filtered by age or skill level. The child may stand or sit, or difficulty could further be graded with the addition of therapy equipment, such as sitting on a therapy ball or standing on a rocker board. At the beginning of each session, the software “tracker” will calibrate to identify and recognize the objects to be used. The aide may move the child closer or farther away from the webcam, or adjust the parameter boarders, based on the child’s ability to “reach” to the top of the screen in the virtual space. The supervising aide then selects the games according to the skill-level of the child. For assessment purposes, therapists may track and record progress from session to session via Timocco’s tracking module.

## PROCEDURES

The research team introduced the Timocco program to the school, one year prior to the initiation of the research study. At that time, all teachers and staff received virtual training from Timocco representatives. Teachers and staff had the opportunity to informally utilize Timocco for all children throughout the year. Prior to initiation of the study, Timocco representatives provided further training to the researchers and research assistants. The research team met with the staff at the school to address scheduling, space, and supervision requirements to complete the research study utilizing the program.

Upon the school’s approval, the licensed OT faculty researchers requested an in-person meeting with the potential child participants’ parents. The parents signed consent forms for their children. The children signed assent forms after the purpose, benefits, and risks were explained by the OT researcher. The researchers then reviewed academic records and prior OT consultations and evaluations.

The researchers administered the Bruininks-Oseretsky Test of Motor Proficiency Short Form (BOT2-SF) (Bruininks & Bruininks, 2005) to identify each participant’s gross and fine motor skill abilities. While the OT faculty member administered the assessment, one to two entry-level doctorate occupational therapy students were present observing the child to increase reliability of the scoring.

Under the guidance of the OT faculty researchers, research assistants (two entry-level doctorate occupational therapy students) customized the games and calibrations to meet the needs and preferences of the children. To facilitate participation and maintain consistency from session to session, the research assistants were present on-site during all sessions to monitor, encourage, and grade the difficulty of exercises, as needed. They modeled the role of school staff (simulated the “e-helper” role) to explore feasibility of the consultative telerehabilitation model. The OT researchers were not present for any sessions; however, they could asynchronously monitor the progress of each child by remotely consulting with the assistants, weekly.

The location interchanged between two settings, either within the child’s classroom, or at a “learning station” within a private room. This enabled the researchers to meet the class, children’s, and teachers’ scheduling needs for the time and day.

## INTERVENTION DURATION

Each participant received 20 sessions of Timocco, with sessions lasting 30 minutes, completed within 6 weeks. Generally, the frequency of sessions was 3–4 times per week, to allow for scheduling conflicts related to the participants’ absences from school, due to illness or the school’s spring break. None of the child participants received therapy outside of this study, at the time of the Timocco intervention sessions. The BOT2-SF was administered by an OT after 20 sessions of Timocco, as well as brief, structured interviews with the research assistants and the children.

## ANALYTIC METHODS

A mixed methods design requires rigorous procedures and connection of results for proper interpretation of the data (Creswell, Klassen, Clark & Smith, 2011). Descriptive quantitative data resulted from analysis of pre and post assessment data of the BOT2-PF scores for both children. Inter-rater reliability checks occurred during evaluations by one occupational therapist and two research assistants. All raters observed the child’s performance. While one rater assessed the child, the process attempted to increase reliability during scoring as shared interpretations to identify an accurate score.

**Figure 2:**
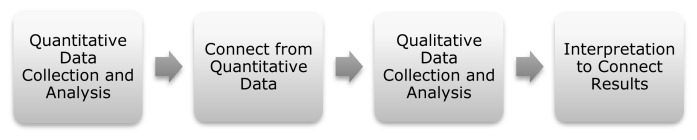
Sequential explanatory design. This form of mixed methods outlines a process starting with quantitative data collection and analysis to then inform the development of questions for interviews directly connecting to the qualitative methods (Creswell, Klassen, Clark & Smith, 2011).

**Figure 3:**
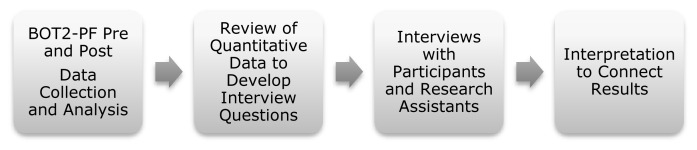
Use of sequential explanatory design for the current project.

A sequential explanatory design (Figure 2) guided the mixed methods approach. The results from the post assessments informed the development of interview questions (Figure 3). For the qualitative data, child and research assistant (OT students) interviews occurred. One faculty researcher interviewed each person privately at the school; the same researcher transcribed the responses. Qualitative data was analyzed independently by the other faculty researcher who did not complete the interviews, to identify themes in the data.

## RESULTS

Pre and post assessment scores from the BOT2-SF revealed an increase in the overall scores for both children, as described in Table 2. Sub-scores for both children are outlined in Table 3. Child #1 improved in five out of the eight sub-tests. The areas of *Fine Motor Precision* and *Balance* did not change. However, *Strength* decreased from a score of four to two. Child #2, improved in three out of the eight areas. The other areas remained the same with the exception of *Running Speed and Agility* and *Upper-Limb Coordination*, showing slightly decreased scores.

## OUTCOMES

The quantitative data unveiled an overall positive experience supporting the usage within the setting. However, there were areas lacking improvement. We further explored these results and reviewed the feasibility through the qualitative interviews performed (Table 4). Both children reported positive experiences with the Timocco program. Yet, they both shared concerns.

### Child #1

*Interviewer:* “What did you like about Timocco?”

“It was so fun.” “I liked the dressing game the most because he gets to go to school.” “I like to do Timocco sitting” [versus standing].

*Interviewer:* “What didn’t you like about Timocco?”

“The space game. Too many aliens and I don’t like aliens, they give me nightmares.”

“I don’t like that it sticks” [time it takes to calibrate].

*Interviewer:* “Did you get to do Timocco in your class?”

“Did it in and out of class. I liked it better to go outside of class with my teachers” [student researchers].

### Child #2

*Interviewer:* “What did you like about Timocco?”

“My favorite part about it is that I like that you get to do easy activities and that it doesn’t get too hard for you.”

*Interviewer:* “What didn’t you like about Timocco?”

“I do like it, but I would maybe change how you use the balls” [needing to use the motion capture red and blue balls]. “Cookie mania; if the balls were not calibrated right, I couldn’t get the cookie where I wanted it to go.”

*Interviewer:* “What did you like best about Timocco?”

“It helped me with my eye-hand coordination.” “I like to do it standing.”

### FEASIBILITY

One researcher asked the two assistants to freely share their experiences of using the Timocco program at the end of the project, within an unstructured interview. They reported the following which was transcribed by the same researcher:

#### RESEARCH ASSISTANT #1

“The dressing game can be memorized” [why Child #1 liked the dressing game the best]. “I feel like if I didn’t have an OT background, it would be difficult to grade the tasks. You can only do so many things to grade it because you have to stay in front of the screen. One student would get really bored, but the other student could sit there. You could get a good 10–15 minutes of attention out of the second student, then he would want to move around.”

“Younger students need more one-on-one attention. The teachers need something that they can just leave the kid to do on their own; the kids play what they want to play, but they don’t play what they need to improve.”

#### RESEARCH ASSISTANT #2

“I liked that there were a lot of options to work on the same goals, so they don’t get bored. We were able to adjust the grading of tasks, and it’s easy to find a game that works when there are so many.”

“I don’t think that parents and teachers can independently think of how to grade the activity” [without OT assistance].

“30 minutes continuously of Timocco is a little unrealistic. We could only keep attention by consistently changing the games. 10–15 minutes at a time is more realistic.”

## DISCUSSION

Timocco proved positive for feasibility in implementation in the private school setting with “at-risk children.” The quantitative data indicates the usefulness of the intervention in enhancing underlying skills required for school. Furthermore, the qualitative data provides further insight in the administering, and ease of use, of the Timocco program in this unique school setting.

Both children improved motor skills as measured on the BOT2-SF and anecdotal verbal reports of the school staff indicated enhanced school performance as a result of Timocco use. Both children also improved in the area of fine motor integration. These results were perhaps related to the OT based intervention activities selected. The qualitative reports assisted in gaining further understanding of the quantitative data. Yet, there are areas for both children that did not improve, or revealed decline.

Child #1 showed gains in the majority of the sub-tests. Yet, his scores for fine motor precision, balance, and strength did not improve. To this end, the quantitative data may further be explained in the reported qualitative interviews revealing his desire to perform a specific game. That the selected game addressed motor control, accuracy, and sequencing indicates a relation to the areas improved. Conversely, the quantitative data revealed decreased strength and lack of improvement in balance. Again, the qualitative data provided possible insight as the child desired to sit during the activities versus standing. Additionally, Research Assistant #1 indicated the child’s desire to play one specific game perhaps limiting his experience and challenges in the other areas.

Child #2 showed overall gains with minimal improvement in the sub-tests. His reporting of the easiness of the games could be an indication of why his scores did not greatly improve. The Research Assistant #1 shared that the child would lose focus and desired to move around. Hence, his distraction could have impeded his success.

Due to lack of formal follow up data, the researchers can only assume a relationship between the Timocco intervention and the two children’s school-based performance. Nevertheless, OT services utilize evidence-based practice, and the skills addressed in the Timocco program address areas of performance required in academic grade school settings.

### BARRIERS

Within the private scholarship-based school setting, teachers and staff instruct a rigorous curriculum. The school implements a schedule that does not easily accommodate the inclusion of any additional services.

Because the research assistants were doctoral OT students, the exploration of the feasibility of using routine school staff to implement the consultative model is not generalizable.

Timocco allows therapists to review data, update programs, and develop goals through a virtual, distance platform. A full utilization of these features was lacking in this study due to the focus on the introduction and implementation of the program at the setting.

The researchers and research assistants resolved initial technical concerns such as how to access the program, availability of school computers, and identifying appropriate time frames for program implementation during the school day.

### SUGGESTIONS FOR FUTURE USE

Maintaining attention is a consideration when working with children with various developmental needs. Therefore, it should be clarified that when using any technological platform, close supervision for cueing, encouragement, and guiding is required for child participation. And, classroom staff needs ongoing training and support to ensure the sustainability of online services.

Based upon the results, the OT needs to identify specific games based on each child’s needs. While the researchers initially performed this step, revisiting the games following a review of the child’s performance could have enhanced the outcomes. For future use, the OT should monitor a child’s performance and goals weekly, and revise activities as needed. Therefore, we recommend use of the Timocco program as an adjunctive therapy, not as a replacement for OT services.

## LIMITATIONS

The study design and small sample size limits the generalizability of the findings. A larger pilot study is necessary to determine the appropriate sample size for a randomized trial, including a control group that is not receiving adjunctive services via telerehabilitation.

Additionally, to maintain privacy of this “at risk” population, we did not obtain health or diagnostic records. Therefore, we were unable to examine the background or underlying performance skills that the child may lack due to a medical diagnosis, as well as infer if additional practice with motor-based exercises are beneficial for a child with a specific diagnosis. In the future, we plan to recruit more homogeneity in the sample to control for diagnosis and attention to task, recommending a range in BOT2-SF scores for inclusion.

A final limitation of this study is that the application of the Timocco program was enhanced by occupational therapy-related input. The research assistants were entry-level doctorate occupational therapy students completing their final semester of their studies. For example, we assume that the concept of “upgrading” or “downgrading” of treatment was implemented by the research assistants, possibly simulating what a licensed OT practitioner may do in a therapy session. This is consistent with previously published literature, that the simulation of therapist assistance may provide an added benefit (Ingersoll, Wainer, Berger, Pickard, & Bonter, 2016). This limits the generalizability that this “just right challenge” could be replicated by school staff or parents within the child’s environment.

## CONCLUSION

The primary purpose of this study was to explore the feasibility of Timocco use in a school-based setting. A secondary purpose was to explore the feasibly of Timocco use via the qualitative verbal reports of the research assistants and the child participants. Overall, the children and research assistants reported positive experiences with the use of this motion-capture platform. Furthermore, Timocco proved positive for feasibility in implementation in a school setting with “at-risk” children.

For children, frequent telerehabilitation-based skill practice using a motion-capture platform such as Timocco may yield promising results to promote motor skill improvements.

The findings of these mixed methods case reports suggest the potential for motion-capture platforms to be used as a successful delivery method for adjunctive practice with children, in a school-based setting. A collaborative, consultative model is recommended with frequent input from the child, aides or teachers, and therapy practitioners. Therefore, we recommend these sessions as adjunctive to regular occupational therapy services to enhance improvement, not as a replacement for stand-alone evaluations and treatment.

## Figures and Tables

**Table 1:** Characteristics of the Child Participants

Characteristics	Child #1	Child #2
Gender	Male	Male
Age (years)	5	7
Race	African American	Caucasian
The Bruininks-Oseretsky Test of Motor Proficiency Short Form (BOT2-SF), at baseline (x/88)	34	55

**Table 2:** Bruininks-Oseretsky Test of Motor Proficiency Short Form (BOT2-SF) Pre and Post Scores

BOT2-SF (x/88)	Child #1	Child #2
Pre Intervention March 15, 2016	34	55
Post Intervention April 26, 2016	46	58
Increase in Score	12 points	3 points

**Table 3:** The Bruininks-Oseretsky Test of Motor Proficiency Short Form (BOT2-SF) Detailed Sub-Scores

	Sub-Test	Pre	Post

**Child #1**	1. Fine Motor Precision	5	5
	2. Fine Motor Integration*	6	8
	3. Manual Dexterity*	3	4
	4. Bilateral Coordination*	5	6
	5. Balance	8	8
	6. Running Speed and Agility*	3	4
	7. Upper-Limb Coordination*	0	9
	8. Strength	4	2

**Child #2**	1. Fine Motor Precision*	7	9
	2. Fine Motor Integration*	8	10
	3. Manual Dexterity	5	5
	4. Bilateral Coordination	6	6
	5. Balance	8	8
	6. Running Speed and Agility	9	8
	7. Upper-Limb Coordination	8	6
	8. Strength *	4	6

Note.

* indicates areas of improvement

**Table 4:** Connection of Quantitative to Qualitative Results

Connection of Results	Sub-Test Mean Quantitative Scores	Qualitative Report
Child #1 Areas of Improvement	Fine Motor Integration^*^ Manual Dexterity^*^ Bilateral Coordination^*^ Running Speed and Agility^*^ Upper-Limb Coordination^*^	Enjoyed dressing game: “What’s Next” (motor control and accuracy, teamwork, sequencing, and planning in a daily context)
Child #1 Areas Lacking Improvement	Fine Motor Precision Balance Strength	Dislike of aliens in game “Aliens in Space” (motor control, accuracy and efficiency, bilateral coordination, attention skills, inhibition and shifting) Preferred Sitting
Child #2 Areas of Improvement	Precision^*^ Fine Motor Integration^*^ Strength ^*^	Helped with eye- hand coordination Enjoyed playing in standing
Child #2 Areas Lacking Improvement	Fine Motor Manual Dexterity Bilateral Coordination Balance Running Speed and Agility Upper-Limb Coordination	Games were easy activities: “Cookie Mania” (motor control, accuracy and efficiency coordination attention skills) did not work appropriately
